# Mechanisms of Vitamins Inhibiting Ferroptosis

**DOI:** 10.3390/antiox13121571

**Published:** 2024-12-20

**Authors:** Meng Zhang, Xin Chen, Yumei Zhang

**Affiliations:** 1 College of Veterinary Medicine, Yangzhou University, Yangzhou 225009, China; dz120210022@stu.yzu.edu.cn (M.Z.); xinchen@yzu.edu.cn (X.C.); 2 Jiangsu Co-Innovation Center for Prevention and Control of Important Animal Infectious Diseases and Zoonoses, Yangzhou 225009, China; 3 Joint International Research Laboratory of Agriculture and Agri-Product Safety, the Ministry of Education of China, Yangzhou University, Yangzhou 225009, China

**Keywords:** vitamins, ferroptosis, lipid peroxidation, cell death

## Abstract

Ferroptosis is an iron-dependent form of cell death, which is characterized by the uncontrolled and overwhelming peroxidation of cell membrane lipids. Ferroptosis has been implicated in the progression of various pathologies, including steatotic liver, heart failure, neurodegenerative diseases, and diabetes. Targeted inhibition of ferroptosis provides a promising strategy to treat ferroptosis-related diseases. Multivitamins, including vitamins A, B, C, D, E, and K, have shown a good ability to inhibit ferroptosis. For example, vitamin A significantly upregulated the expression of several key ferroptotic gatekeepers genes through nuclear retinoic acid receptors and retinoic X receptors (RAR/RXR). Vitamin B6 could compensate for the impaired glutathione (GSH) levels and restore Glutathione peroxidase 4 (GPX4) expression in cells, ultimately inhibiting ferroptosis. Vitamin D could up-regulate the expression of several anti-ferroptosis proteins by activating vitamin D receptors. Vitamin E and hydroquinone vitamin K (VKH_2_) can directly inhibit the propagation of lipid peroxidation, thereby inhibiting ferroptosis. In this review, we summarize the currently understood mechanisms by which vitamins inhibit ferroptosis to provide reference information for future research on the development of ferroptosis inhibitors.

## 1. Introduction

Ferroptosis is a non-apoptotic form of programmed cell death that is initiated by the overwhelming accumulation of iron-dependent membrane lipid peroxides [1]. Although specialized forms of ferroptosis-like cell death were reported long before, they are not integrated into a unified process. For instance, Harry Eagle’s pioneering work in the 1950s revealed that the deprivation of the amino acid cysteine resulted in cell death. Additionally, in 2003, a small molecule known as erastin was reported to selectively induce tumor cell death through non-apoptotic mechanisms [2]. In 2008, inducible Glutathione peroxidase 4 (GPX4) inactivation in mice and cells revealed a new form of oxidative cell death, distinct from apoptosis, which can be prevented with vitamin E [3]. Ferroptosis was first proposed by Dixon et al. in 2012 and is now appreciated as likely one of the most widespread and ancient forms of cell death [1].

Ferroptosis is regulated by multiple cellular metabolic pathways, including redox homeostasis, iron, mitochondrial activity, and the metabolism of amino acids, lipids, and sugars, along with various signaling pathways associated with disease [4]. Mounting evidence suggests numerous organ injuries and degenerative pathologies are driven by ferroptosis, including aging, nervous system diseases [5], kidney disorders [6], fatty liver [7], and osteoporosis [8]. Targeted inhibition of ferroptosis has shown excellent protective and therapeutic effects in various animal disease models [9,10]. Therefore, inhibiting ferroptosis might represent a promising strategy to treat related diseases [9,10,11]. Significant research effort and financial resources have been invested to discover or modify drug-like candidates as ferroptosis inhibitors to treat ferroptosis-driven diseases [11]. However, pharmaceutical agents aimed at inhibiting ferroptosis have not been applied clinically because of problems related to their bioavailability, solubility, and toxicity [11]. In contrast, vitamins, which are essential micronutrients crucial to maintain normal physiological functions, have a lower toxic effect and a wider range of sources. Additionally, some vitamins exhibit potential anti-ferroptosis abilities [12,13,14,15,16,17]. In this review, we summarize the mechanisms of vitamins, including vitamin A, B, C, D, E and K, in inhibiting ferroptosis and discuss the potential of vitamins to treat or alleviate ferroptosis-related diseases. Our summary will provide important reference information for future research on the development of ferroptosis inhibitors and therapies for ferroptosis-related diseases.

## 2. Ferroptosis

Morphologically, cells undergoing ferroptosis exhibit dysmorphic small mitochondria and a decreased number of cristae, which contrasts with other forms of cell death [18]. Mechanistically, although the underlying processes remain largely elusive compared with other forms of cell death, a complex regulatory network for ferroptosis has been established [10]. The accumulation of iron, the overproduction of reactive oxygen species (ROS), and the synthesis of oxidized phospholipids (PLs) containing polyunsaturated fatty acids (PUFA-PLs) are essential for the lipid peroxidation that drives ferroptosis [10]. In the following sections, we summarize the primary mechanisms currently known to regulate ferroptosis, forming targets to induce resistance or inhibition.

### 2.1. Drivers of Ferroptosis

Unrestrained lipid peroxidation, the hallmark of ferroptosis, can be divided into three phases: initiation, propagation, and termination [19]. The initiation lipid peroxidation requires the formation of phospholipid radicals (PL·), which are produced by PL under enzymatic and Fenton reactions. In propagation, PL· reacts with molecular oxygen to form a phospholipid hydroperoxide radical (PLOO·), which removes hydrogen from another PUFA, forming lipid peroxides (PLOOH) [20]. Subsequently, PLOOH and lipid free radicals will continue to dehydrogenate with PL and react with molecular oxygen to form PLOOH, resulting in the excessive production of PLOOH. Eventually, this results in the formation of various secondary products, such as 4-hydroxynonenal and malondialdehyde, as well as breakdown products of oxidized and modified proteins [19].

PUFA-PLs is synthesized by PUFAs under the action of a series of enzymes and incorporated into lipid bilayers. Among PUFAs, omega-6 and omega-3 PUFAs, named for the position of the most terminal double bond on the acyl chain, are essential and the most abundant cellular PUFAs [21]. Eicosatetraenoic acid (EPA) and docosahexaenoic acid (DHA) are important omega-3 PUFAs, while linoleic acid (LA) and arachidonic acid (ARA) are key omega-6 PUFAs. They play significant roles in various physiological and pathological functions, including inflammation, oxidation, and nervous system development [21,22]. E-series resolvins (RvEs) synthesized from EPA, D-series resolvins (RvDs), and protectins (PDs) derived from DHA are the primary anti-inflammatory lipid mediators [21]. Numerous prospective studies have demonstrated associations between dietary or circulating levels of EPA and DHA and the risk of coronary artery disease (CAD) [23,24,25]. Individuals with higher levels of EPA and DHA in their blood exhibit a significantly reduced risk of CAD. Furthermore, various clinical studies have indicated that high-dose treatment with EPA and DHA markedly slows the progression of CAD in patients [25]. The role of omega-6 PUFAs remains a subject of debate. ARA serves as a precursor for several pro-inflammatory mediators, including prostaglandins and leukotrienes [21]. The development of anti-inflammatory drugs targeting the ARA pathway has proven effective in managing inflammation. Additionally, LA is the direct precursor of bioactive oxidized linoleic acid metabolites [26]. In mammalian cells, peroxidation of omega-6 PUFAs is strongly associated with ferroptosis [27]. However, intake of LA or ARA does not increase the concentrations of various inflammatory markers [26].

**Figure 1:**
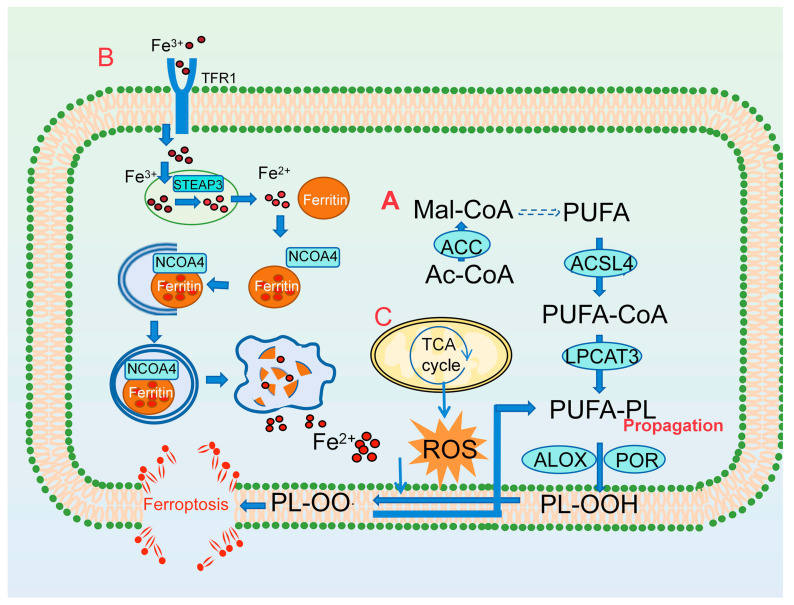
The driver mechanisms of ferroptosis. (**A**) The PUFA-PL synthesis pathway. (**B**) The iron metabolism pathway. (**C**) Reactive oxygen species (ROS). ACSL4, acyl-CoA synthetase long-chain family member 4; LPCAT3, lysophosphatidylcholine acyltransferase 3; ACC, acetyl-CoA carboxylase; ALOX, arachidonate lipoxygenase; POR, cytochrome P450 oxidoreductase; PL-OOH, lipid peroxides; PL-OO·, alkoxyl and peroxyl radicals; TFR1, transferrin receptor 1; STEAP3, six-transmembrane epithelial antigens of prostate 3; NCOA4, nuclear receptor activator 4; Mal-CoA, malonyl-coenzyme A; PUFA, polyunsaturated fatty acid; Ac-CoA, acetyl-coenzyme A.

The presence of weak C–H bonds at the bis-allylic positions in PUFAs in which ARA (20:4) and adrenalinic acid (AdA (22:4)), oxidized forms of two omega-6 PUFAs, are the primary substrates for lipid peroxidation in ferroptosis make them highly susceptible to oxidation [10]. These PUFAs are synthesized primarily from malonyl-CoA, which is produced from acetyl-CoA under carboxylation by acetyl-CoA carboxylase (ACC) (Figure 1A) [28]. Therefore, ACC is necessary for ferroptosis. Notably, peroxidation of free PUFAs does not directly trigger ferroptosis. The esterified membrane PLs form free PUFAs, which directly trigger ferroptosis [29]. Acyl-CoA synthetase long-chain family member 4 (ACSL4) ligates PUFAs with CoA to produce PUFA-CoA, and then these products are esterified onto PLs by various lysophosphatidylcholine acyltransferases (LPCATs) in the cell membrane [29]. Membrane lipid peroxidation is driven by enzymatic reactions and the Fenton reaction. Arachidonate lipoxygenases (ALOXs) and cytochrome P450 oxidoreductase (POR) play a primary role in the enzymatic reaction of lipid peroxidation, which can catalyze the peroxidation of PUFA-PLs to generate PL-OOH [4]. In the non-enzymatic lipid peroxidation carried out using the Fenton reaction, PL-OOH interacts with ferrous irons and superoxide ions (a kind of ROS) to PL-OO·, consequently leading to the propagation of PL-OOH production. The propagation reaction driven by PL-OO· is dominant at the initiation phase of ferroptosis [30,31]. This chain reaction leads to the breakdown of membrane integrity and causes cell membrane damage, ultimately leading to cell death (Figure 1A).

As is evident from the name itself, ferroptotic cell death depends on iron. A non-enzymatic, iron-dependent Fenton reaction may be necessary for ferroptosis. Iron commonly exists in one of two oxidation states: the oxidized form, Fe^3+^; or the reduced form, Fe^2+^ [19]. Fe^3+^, the primary form of iron in circulation, is delivered into cell endosomes with the assistance of transferrin receptor 1 (TFR1) [32]. In endocytosed vesicles, Fe^3+^ is reduced to Fe^2+^ by six-transmembrane epithelial antigens of prostate 3 (STEAP3) and then released into the cytoplasm with the aid of the divalent metal transporter 1 (DMT1), forming the labile iron pool [33]. Excess Fe^2+^ in the labile iron pool can be conveyed to the extracellular space by membrane iron transporter 1 or stored in ferritin, consisting of ferritin heavy chain 1 (FTH1) and ferritin light chain (FTL), to maintain intracellular iron homeostasis [34]. Ferritin was discovered by Laufberger in the 1930s [35]. Initially, scientists thought that ferritin could promote lipid peroxidation, primarily due to the experimental conditions at the time [36,37,38,39]. They observed that ferritin could enhance lipid peroxidation in liposomes and microsomes in vitro. This effect may be attributed to ferritin increasing bivalent iron ion levels in the reaction system, rather than to ferritin itself [40,41]. In contrast, subsequent studies found that ferritin protects against endothelial cell lysis induced by hydrogen peroxide through the chelation of ions [42]. Since that time, an increasing number of studies have reported the anti-lipid peroxidation effect [43,44]. Following the proposal of the concept of ferroptosis by Doxin in 2012, ferritin garnered significant attention. Fang et al. found that genetic deletion of the ferritin heavy chain promotes cardiomyopathy, likely via enhancing ferroptosis [45]. Additionally, increasing cellular iron availability by autophagic degradation of the ferritin promotes ferroptosis [46]. Ferritinophagy regulated by nuclear receptor activator 4 (NOCA4) increases the size of the labile iron pool, resulting in iron overload, which triggers ferroptosis (Figure 1B) [47,48].

In addition, ROS, mainly produced by the electronic transport chain (ETC) in mitochondria, are essential for the accumulation of lipid peroxides (Figure 1C). Various antioxidant compounds have been found to inhibit ferroptosis.

### 2.2. Defense Against Ferroptosis

To date, various anti-ferroptosis systems have been identified that inhibit ferroptosis, mainly by reducing PL-OOHs to PL-OH or PL-OO· to PL-OOH [4].

#### 2.2.1. The Glutamate Antiporter System Xc−

**Figure 2:**
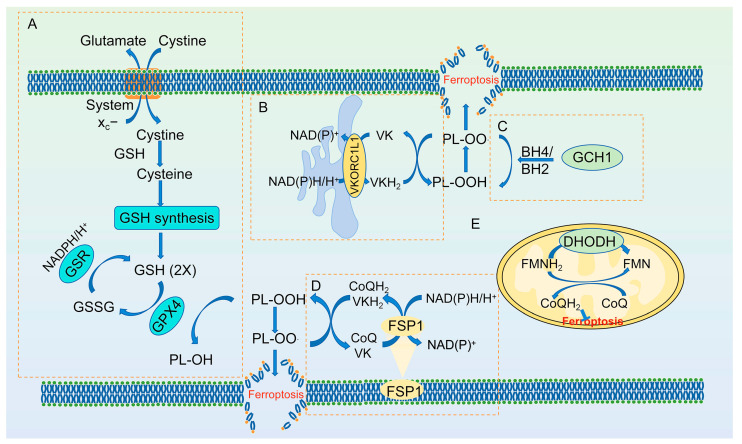
The defense mechanisms against ferroptosis. (**A**) The Xc−/GSH system. The anti-ferroptosis mechanisms of VKORC1L1 (**B**); GCH1 (**C**); FSP1 (**D**); DHODH (**E**). GPX4, glutathione peroxidase 4; glutathione (GSH); VKORC1L1, vitamin K epoxide reductase complex subunit 1 like 1; FSP1, ferroptosis suppressor protein 1; DHODH, dihydroorotate dehydrogenase; GSR, glutathione reductase; GSSG, glutathione disulfide; VK, vitamin K; BH2, tetrahydrobiopterin; BH4, dihydrobiopterin; GCH1, GTP Cyclohydrolase 1; FMN, flavin mononucleotide; FMNH_2_, reduced flavin mononucleotide.

GPX4, a selenoprotein, exhibits a strong capacity to resist ferroptosis [49]. It catalyzes the reduction of PL-OOHs to PL-OH, thereby limiting the propagation of lipid peroxidation within cellular membranes. This reduction reaction requires the catalytic selenocysteine residue of GPX4 and two electrons, which are typically supplied by glutathione (GSH) [50]. The tripeptide GSH is derived from cysteine, glutamic acid, and glycine, among which cysteine is the rate-limiting precursor and is rapidly converted from cystine within cells. The GSH antiporter system Xc−, comprising cystine/glutamate reverse transporter solute carrier family 3 member 2 (SLC3A2) and cystine/glutamate reverse transporter solute carrier family 7 member 11 (SLC7A11), mediates the exchange of intracellular glutamate for extracellular cystine, ensuring an adequate supply of cystine for cells [51]. Consequently, the inactivation of GPX4 or a deficiency in GSH hampers the timely clearance of lipid peroxides, leading to protein and membrane damage, ultimately resulting in ferroptosis (Figure 2A). Erastin and RAS-selective lethal 3 (RSL3), two common inducers of ferroptosis, inhibit system Xc− and GPX4 activity, respectively, to induce ferroptosis [49].

#### 2.2.2. Vitamin K Epoxide Reductase Complex Subunit 1 Like 1 (VKORC1L1)

The vitamin K oxidoreductase VKORC1L1 is expressed primarily in the endoplasmic reticulum [52]. As early as 2011, Westhofen et al. reported that VKORC1L1 can reduce intracellular ROS levels and oxidative damage to membrane proteins [53]. Recently, VKORC1L1 has been identified as an anti-ferroptosis protein. Mechanistically, VKORC1L1 reduces vitamin K to hydroquinone vitamin K (VKH_2_), an effective radical-trapping antioxidant (RTA) that eliminates lipid peroxides and reduces PL-OO· to PL-OOHs, thereby inhibiting ferroptosis (Figure 2B) [54]. Furthermore, the expression of VKORC1L1 is directly regulated by P53. Activation of P53 decreases VKORC1L1 expression, increasing cell susceptibility to ferroptosis [54]. Additionally, warfarin, a VKORC1L1 inhibitor, causes ferroptosis in tumors with a high level of VKORC1L1 expression [54].

#### 2.2.3. FSP1

Ferroptosis suppressor protein 1 (FSP1) is a member of the type II nicotinamide adenine dinucleotide-H (NADH): quinone oxidoreductase (NDH-2) family [55]. It is also known as apoptosis-inducing factor mitochondria associated 2 (AIFM2) because of its structural homology with apoptosis-inducing factor (AIF), and is located in the cell membrane and ER [55]. This enzyme possesses coenzyme Q10 (CoQ10) oxidoreductase and vitamin K reductase activity and can reduce CoQ10 or vitamin K to produce CoQ10H_2_ and VKH_2_ in an NADPH-dependent manner [55,56]. These products can suppress the superoxide radical deriving propagation reaction of PL, thereby inhibiting ferroptosis (Figure 2D) [56]. Inhibitor iFSP1 treatment or *Fsp1* gene deletion can promote ferroptosis.

#### 2.2.4. GTP Cyclohydrolase 1 (GCH1)

Biosynthesis of tetrahydrobiopterin (BH4) from its precursor GTP requires three enzymatic steps catalyzed by GCH1, 6-pyridoxyl tetrahydropterin synthase (PTS), and sepiapterin reductase (SPR), with GCH1 serving as the rate-limiting enzyme [57]. Genetic or pharmacological suppression of GCH1 results in BH4 deficiency, leading to the accumulation of peroxides and subsequent cellular ferroptosis [58]. Dihydrobiopterin (BH2) interacts with BH4 to establish a cycle that reduces endogenous oxidizing free radicals and protects lipid membranes, thereby inhibiting ferroptosis (Figure 2C). The regeneration of BH4 from BH2 is mediated by dihydrofolate reductase (DHFR), which utilizes NADP/NADPH as a cofactor [59]. Notably, supplementation with BH4, but not BH2, effectively reversed DHFR inhibitor-induced ferroptosis in GCH1 knockout cells.

#### 2.2.5. DHODH/CoQ

Dihydroorotate dehydrogenase (DHODH) is an flavin-dependent enzyme localized in the mitochondrial inner membrane, playing an important role in pyrimidine synthesis. The enzyme reduces ubiquinone (CoQ) to ubiquinol (CoQH_2_) to detoxify lipid peroxides in mitochondria (Figure 2E) [60]. Targeted inhibition of DHODH-mediated ferroptosis defense is a strategy used to treat cancer [60,61].

## 3. Vitamins and Ferroptosis: Mechanisms and Treatment

### 3.1. Vitamin A

Vitamin A, a fat-soluble essential vitamin, refers to a class of compounds characterized by unsaturated isoprenoid chain structures, including retinol, retinyl ester (RE), carotenoids, and retinoic acid (RA, the biologically active form of vitamin A) [62,63]. In the daily diet, vitamin A is ingested mainly in the form of REs (from animal foods) and carotenoids (from plant foods) [64]. The facile endogenous hydrolysis of REs forms retinol in the gut. Carotenoids and REs are incorporated into mixed micelles along with other lipids and are absorbed by intestinal epithelial cells. Retinol absorption efficiency is higher than that of beta-carotene, which might be caused by the presence of retinol-specific receptors in the gut. After absorption by the gut, retinol is esterified to REs and transported to the liver as part of chylomicron residues with carotenoids. Within the liver, REs are hydrolyzed back to retinol and carotenoids are metabolized into retinal, mediated by beta-carotene 15,15′-monooxygenase enzyme (BCO1), and can then be converted to retinol [63,64,65]. Subsequently, retinol and retinol-binding protein (RBP4) are released from the liver and transported to other tissues outside the liver for the synthesis of intracellular RA [64,65]. Indeed, RA plays an important role in diverse biological processes, including embryonic development, immune function, reproduction, and neurodevelopment, by binding the nuclear retinoic acid receptors (RARs), retinoic X receptors (RXRs), and polymorphic retinoic acid response elements (RAREs) within the promoter regions of responsive genes to regulate gene expression [66,67,68,69]. Vitamin A deficiency is the most common nutritional disorder in developing countries, resulting in dry eyes and blindness, as well as increased risk of death from infections [70].

**Figure 3:**
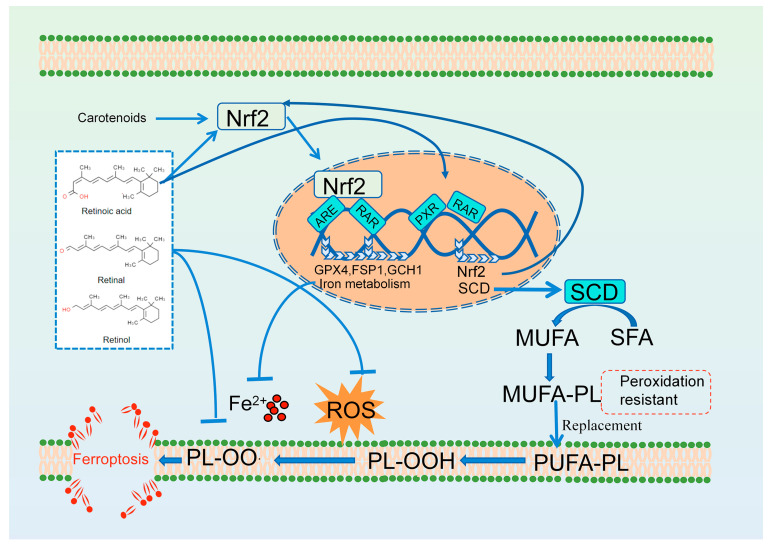
The anti-ferroptosis mechanism of vitamin A. RAR, retinoic acid receptor;; SCD, stearoyl-CoA desaturase; NRF2, nuclear factor erythroid 2-related factor 2; ARE, acid response element; MUFA, monounsaturated fatty acid; SFA, saturated fatty acid.

Ferroptosis is triggered upon lipid peroxidation. As early as the 1970s, vitamin A (carotenoids) was found to prevent lipid peroxidation [71]. In addition, vitamin A at pharmacologic doses ameliorates lipid peroxidation injury in rats caused by alcohol, cigarettes, and many medications [72,73]. With our deepening knowledge of ferroptosis, vitamin A has been found to inhibit ferroptosis through a variety of mechanisms. Jakaria et al. revealed that vitamin A and its metabolites all-trans-retinal (atRAL) and all-trans-retinoic acid (atRA) exhibited higher anti-ferroptotic abilities (retinol EC_50_: 0.4–4.8 μM, atRAL EC_50_: 0.7–1.1 μM and atRA EC_50_: 4.4–11.8 μM) than α-tocopherol (EC_50_: 7.4–36.1 μM) in N27 and HT-1080 cells treated with erastin and RSL3, suggesting that vitamin A has extensive prospects to treat ferroptosis-related disease [74]. Vitamin A (retinol and its metabolites atRAL and atRA) can quench ROS and stabilize peroxy radicals directly because they contain the side chains of polyene units, also known as highly conjugated double-bond systems (Figure 3) [75]. However, the authors proposed an alternative perspective, suggesting that vitamin A does not directly capture free radicals. They stated that the antioxidant properties of vitamin A were demonstrated in solvent and cell culture experiments only at super physiological concentrations; however, there is no convincing in vivo evidence that vitamin A acts as a direct free radical-trapping antioxidant [75]. A recent study suggested that RA exhibits resistance to RSL3-induced ferroptosis in HT-1080 cells by binding to RAR/RXR, rather than through the direct capture of peroxides. In contrast, precursor forms of RA, including retinal, demonstrate the ability to resist ferroptosis by directly capturing anti-peroxides [76]. Treatment with 10 μM RA significantly upregulated the expression of several genes that have been established as encoding key ferroptotic gatekeepers, e.g., *Gpx4*, *Fsp1*, and *Gch1*, through RAR/RXR (Figure 3). In addition, 20 μM RA treatment increased the expression of anti-ferroptosis regulators involved in lipid biosynthesis and lipid metabolism, including acyl-coA synthetase long chain family member 3 (ACSL3) and stearoyl-CoA desaturase (SCD), in HT-1080 cells (Figure 3) [76]. The susceptibility of PUFAs in glycerophospholipids to oxidation suggests them as substrates for ferroptosis. In contrast, monounsaturated fatty acids (MUFAs) in membrane phospholipids are less prone to oxidation, thereby providing protection against ferroptosis. SCD is an enzymatic that converts saturated fatty acids into MUFAs. Bi et al. indicated that 2 μM R regulates the transcription of *Scd* through RARs in A549, HT1080, and H1299 cells, thereby mediating the production of MUFAs [77]. In addition, studies have reported that RA and some non-vitamin A carotenoids (e.g., lycopene, astaxanthin, fucoxanthin, and crocin) prevent ferroptosis in some diseases by promoting nuclear factor erythroid 2-related factor 2 (NRF2) nuclear translocation [78,79,80,81,82]. In cells, NRF2 is the main redox-sensitive transcription factor that responds to oxidative stress, which is translocated to the nucleus and coordinates the expression of a vast array of cytoprotective genes, including anti-redox, autophagy, unfolded protein response, and ferroptosis defense system-related genes (*Slc7a11*, *Gpx4*, *Fsp1*, *Ftl*, *Fth1*, and *Hmox1* (encoding heme oxygenase 1)) [83]. The promoter region of the NRF2 gene contains the peroxisome proliferator response element (PPRE) site, which can interact with the activated peroxisome proliferator activated receptor gamma (PPARγ)-RXR heterodimer [84,85]. These carotenoids, or their metabolites, might mediate signal transduction via RAR/RXR dimers, representing a reasonable explanation for the anti-ferroptosis effect of various carotenoids.

### 3.2. Vitamin B

B vitamins comprise a group of eight water soluble vitamins including thiamine (B1), riboflavin (B2), niacin (B3), pantothenic acid (B5), pyridoxine (B6), biotin (B7), folate (B9), and cobalamin (B12) [86,87]. B vitamins play a crucial role in cellular metabolic processes, serving as cofactors for numerous metabolic enzymes. They are essential for brain function, contributing to energy production as well as DNA and RNA synthesis and repair [86]. Due to the absence of endogenous biosynthetic pathways, humans are unable to synthesize B vitamins directly and must obtain them from plant or animal sources.

B vitamin deficiency has been linked to various health conditions, including obesity, neural tube defects, and stroke, among others [88]. Qin et al. [89] established a model of vitamin B12 deficiency in early Caenorhabditis elegans and discovered that this deficiency leads to increased lipogenesis in adult organisms, particularly concerning endogenous PUFAs. This process induces lipid peroxidation and ferroptosis, which subsequently results in germline defects. Studies have demonstrated a positive association between low levels of B6 and stroke patients [88,90]. It has also been reported that B6 deficiency triggers an adaptive response in mice that enhances the transcription of GCPII via Sp1. The enzymatic cleavage of the neurotransmitter N-acetylaspartylglutamate (NAAG) by GCPII results in the release of glutamate (Glu), which inhibits System Xc-, ultimately leading to ferroptosis [91]. It is noteworthy that several studies have investigated the alleviating effects of vitamin B6 on disorders associated with ferroptosis. Vitamin B6 has been shown to mitigate ferroptosis induced by chronic sleep deprivation in the hippocampus of mice [92]. Chronic sleep deprivation results in the downregulation of xCT, alters GSH levels, and contributes to ferroptosis. Supplementation with vitamin B6 significantly enhances the expression of the key enzyme CBS in the sulfur transfer pathway, thereby compensating for the impaired GSH levels in cells and restoring GPX4 expression, ultimately inhibiting ferroptosis [92]. In addition, vitamin B6 inhibits lipopolysaccharide (LPS)-induced ferroptosis and apoptosis by enhancing the expression of NRF2 and antioxidant enzymes [93].

### 3.3. Vitamin C

Vitamin C (L-ascorbic acid) is an essential micronutrient in primates, including humans, who lack L-gulono-1, 4-lactone oxidase to synthesize it [94,95]. Vitamin C is an important component of many double oxygenase or single oxygenation enzymes’ activities (e.g., dopamine B-monooxygenase or prolyl 4-hydroxylase) in biosynthesis, especially in collagen, carnitine, and hormone synthesis [96,97]. These enzymes all contain Cu^2+^ or Fe^2+^ as a cofactor and tend to oxidize Fe^2+^ to Fe^3+^, rendering the enzymes inactive [95,98]. Vitamin C is a reducing agent in enzymatic reactions that maintains these metals in the reduced state. Vitamin C deficiency can cause scurvy, mainly because of damage to blood vessels caused by defective collagen synthesis [99].

**Figure 4:**
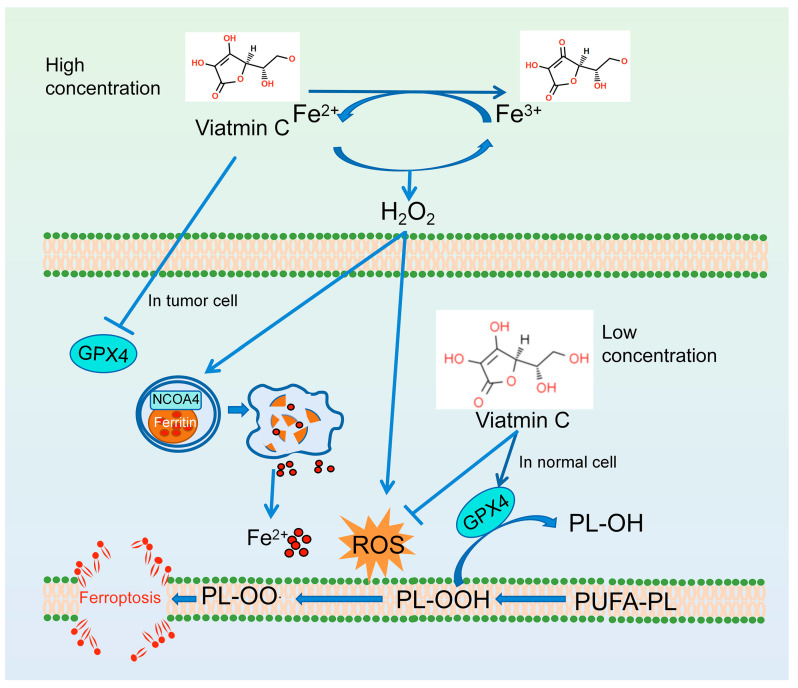
The anti-ferroptosis mechanism of vitamin C.

Vitamin C is a well-known water-soluble antioxidant and free radical scavenger that protects cell components from damage caused by free radicals, which has been clearly and repeatedly demonstrated in vitro [100]. The hydroxyl group located at the double bond in the lactone ring acts as both a proton and electron donor, contributing to the strong reducibility of ascorbic acid [100]. Vitamin C is present at physiological pH in the form of the anion ascorbate. Ascorbic acid acts by donating an electron to neutralize free radicals, forming the ascorbic acid free radical (monodehydroascorbic acid), which then transforms into dehydroascorbic acid. Free radicals can also be converted back into ascorbic acid through enzymatic reactions that depend on NADH or NADPH. In addition, ascorbic acid is essential for the regeneration of other antioxidants, including vitamin E and BH4 [101]. However, the anti-ferroptosis ability of vitamin C has not been widely proven, despite its potent antioxidant properties [102]. One possible explanation is that hydrophilic vitamin C may not have direct contact with lipid free radicals, potentially limiting its ability to neutralize them. It has been suggested that vitamin C has a protective effect against ferroptosis. Research reported that low concentrations of ascorbic acid (0.1–0.5 mM) can avoid the effects of ferroptosis inducers [103]. Vitamin C (150 μL, 1.5 M intraperitoneal injection) has been reported to prevent programmed cell death 1 (PD1) and immunotreatment-induced ferroptosis in hepatocytes by increasing SLC7A11/GPX4 expression in mice; however, this might not be related to the direct antioxidant effect of vitamin C [104]. Conversely, other research suggests that high concentrations of vitamin C might promote peroxidation [103]. In fact, the role of ascorbic acid as a pro-oxidant or antioxidant is thought to depend on its concentration, with low doses (μM) decreasing ROS levels and high doses (mM) producing ROS (Figure 4) [100,102]. Research has demonstrated that intravenous vitamin C can increase the production of hydrogen peroxide in extracellular fluid in a dose-dependent manner [105,106]. Levels of ascorbate free radicals and hydrogen peroxide were elevated in tumors or subcutaneous tissue in mice. The pro-oxidation activity of ascorbic acid is mainly related to the interaction of transition metal ions and copper. Ascorbate can donate electrons to transition metals, leading to the formation of superoxide anions and hydrogen peroxide when the reduced metal reacts with oxygen [100,107]. The reduced iron ions subsequently react with hydrogen peroxide, resulting in cellular damage (Figure 4) [107]. Vaishampayan et al. thought that high doses of ascorbic acid (5–20 mM) kill human osteosarcoma by increasing the surge in cytotoxic ROS, which is iron-dependent, but not via ferroptosis [108]. However, ferroptosis induced by vitamin C has been observed in some tumor cells, as opposed to normal cells, likely resulting from disruptions in iron metabolism and antioxidant systems unique to some tumor cells [102]. In addition, vitamin C has been found to promote ferroptosis in tumor cells by affecting the defense against ferroptosis pathway. The oxidized form of vitamin C, dehydroascorbic acid (DHAA, 1 mM), causes ferroptosis in glioma cells, prostate cancer cells, and tongue squamous cells by affecting GPX4 expression [109]. Wu et al. further revealed that vitamin C can decrease *Gpx4* mRNA levels by affecting signal transduction and transcriptional activator 3 (STAT3) to induce ferroptosis in oropharyngeal cancer [110]. In addition, vitamin C (1 μM in vitro, intraperitoneal injection 4 g/kg in vivo) may increase HMOX1 expression via the AMP-activated protein kinase (AMPK)/NRF2 pathway, leading to elevated levels of labile iron and ultimately increasing pancreatic cancer (PC) cells’ and PC xenografts’ susceptibility to erastin-induced ferroptosis [111]. Furthermore, 1 mM vitamin C treatment induces ferritin autophagy and subsequent ferritin degradation, resulting in the release of free iron, thereby inducing ferroptosis in anaplastic thyroid cancer cells [112].

### 3.4. Vitamin D

Vitamin D, a fat-soluble vitamin, derives from mainly cutaneous7-dehydrocholesterol (7-DHC) (90%) when exposed to solar ultraviolet B (UVB) radiation [113]. Vitamin D from food sources includes vitamin D2 (plants, especially fungi) and vitamin D3 (animals). Vitamin D is transported by binding to the vitamin D binding protein (DBP) to its target organ to complete metabolism and reabsorption [113]. It undergoes hydroxylation to form 25-hydroxyvitamin D (25 (OH) D, circulating form) in the liver, catalyzed by vitamin D 25-hydroxylase (CYP2R1), before being transported to the kidney for further hydroxylation to produce 1,25-dihydroxyvitamin D (1,25-(OH) 2D), catalyzed by 25-OHD-1 alpha-hydroxylase (CYP27B1), which is the active form of vitamin D [114]. Vitamin D deficiency is known to cause osteoporosis and rickets. Infants require a daily vitamin D supplement because of their limited exposure to sunlight and the deficiency of vitamin D in breast milk [115]. In addition, vitamin D plays an important role in maintaining the body’s innate and adaptive immune function, and normal cardiovascular function [116].

**Figure 5:**
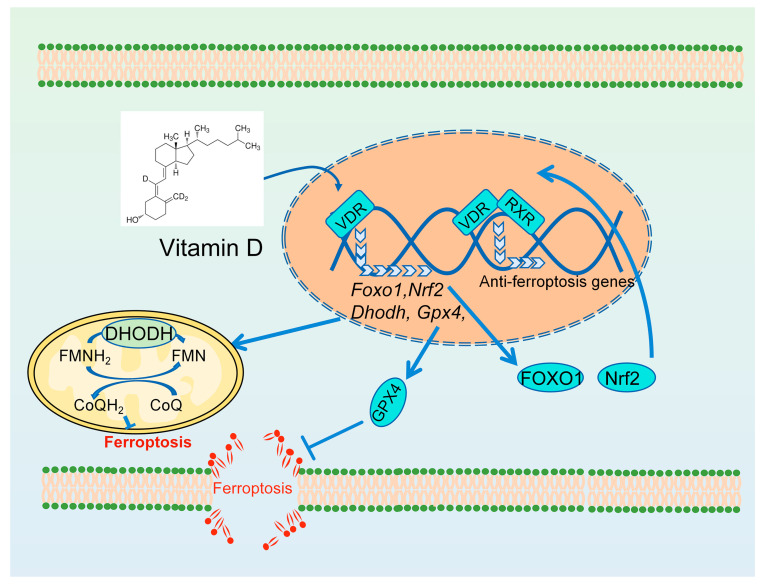
The anti-ferroptosis mechanisms of vitamin D. FOXO1: Forkhead transcription factor.

Hu et al. were the first to report the association between vitamin D and anti-ferroptosis [117]. They found that paricalcitol, an active analog of vitamin D, alleviated cisplatin-induced acute kidney injury in C57/BL6 mice and erastin-induced death in HK-2 cells by activating the vitamin D receptor (VDR), which promotes the transcription of *Gpx4* to inhibit ferroptosis (Figure 5) [117]. Additionally, vitamin D (700 IU/kg/day) mitigates cisplatin-induced intestinal injury by inhibiting ferroptosis through the reduction of iron accumulation and the reversal of GPX4 and DHODH downregulation (Figure 5) [118]. A substantial body of research has demonstrated the occurrence of ferroptosis in various diabetic complications, including diabetes mellitus, diabetic nephropathy [119], and diabetic osteoporosis [120]. Some studies suggested that vitamin D might alleviate diabetes and its associated complications by inhibiting ferroptosis. FOXO1 (encoding Forkhead transcription factor O1, a key regulator in pancreatic beta cells) is a downstream target gene of VDR. Ding et al. reported that vitamin D (0.03 μg/kg BW by gavage) can decrease blood glucose and alleviate pancreatic tissue damage in diabetic rats [121]. 1,25D/VDR inhibited pancreatic β-cell ferroptosis in type 2 diabetes mellitus by downregulating the expression of FOXO1, which decreases ACSL4 levels and increases GPX4 (Figure 5) [121]. Vitamin D supplementation mitigated kidney injury in prediabetic mice by inhibiting ferroptosis via the Klotho/p53 signaling pathway [122]. P53 plays a crucial role in the regulation of ferroptosis by modulating the expression of ferroptosis-related genes, including *Slc7a11* and *Tfr1* [123]. Vitamin D induced resistance to ferroptosis in renal tubular epithelial cells by regulating the NRF2/HMOX1 signaling pathway in diabetic nephropathy [124]. Additionally, vitamin D can alleviate various disease impairments by inhibiting cellular ferroptosis via NRF2. This includes the ferroptosis of osteoblasts in osteoporosis [125] and the ferroptosis of hippocampal neuronal cells in mouse models of cognitive impairment [126], as well as Aeromonas hydrophila-induced ferroptosis in grass carp splenic macrophages [127]. It has been extensively demonstrated that the activation of the VDR can regulate NRF2 transcription or the nuclear translocation of the NRF2 protein (Figure 5) [128,129]. In addition, 1,25(OH)2D3 activated the vitamin D receptor/retinoic X receptor (VDR/RXR) heterodimeric complex in specific DNA sequences [116].

### 3.5. Vitamin E

Vitamin E is naturally synthesized by plants and is predominantly found in seeds, nuts, and vegetable oils. The vitamin E family consists of eight lipophilic antioxidants (four tocopherols and four tocotrienols), including α-tocopherol (αTOH), β-tocopherol (βTOH), γ-tocopherol (γTOH), and δ-tocopherol (δTOH), as well as α-tocotrienol (αTE), β-tocotrienol (βTE), γ-tocotrienol (γTE), and δ-tocotrienol (δTE) [130,131]. All subtypes of vitamin E share a chromanol ring structure with a phenolic group at position 6, which allows its hydrogen atoms to exhibit antioxidant activity [131]. Tocopherols are characterized by a saturated side chain, and tocotrienols have three double bonds in the side chain [132]. All isoforms of vitamin E are biologically active, but only αTOH is retained at high levels in plasma and tissues [132]. Hepatic α-tocopherol transfer protein has higher affinity for αTOH than other isoforms of vitamin E, which protects against excessive degradation and excretion of αTOH [133].

Before the discovery of ferroptosis, vitamin E was recognized as a lipid-soluble antioxidant that effectively eliminates peroxyl radicals and inhibits the propagation of lipid peroxidation [134,135]. As anticipated, vitamin E exhibited significant inhibition of ferroptosis. The anti-ferroptosis properties of vitamin E were first elucidated by Matsushita et al., who demonstrated that vitamin E (αTOH) mitigates T cell ferroptosis induced by the deletion of the Gpx4 gene [136]. Subsequently, the resistance of vitamin E to ferroptosis resulting from GPX4 knockout was confirmed in additional tissues [137,138,139]. Non-alcoholic fatty liver disease (NAFLD) is the most common cause of chronic liver disease and ranges from simple steatosis to non-alcoholic steatohepatitis (NASH) [140]. Currently, the intricate pathophysiology of NAFLD remains poorly understood. While apoptosis and necrosis have traditionally been highlighted, there are presently no specific pharmacological treatments available to reverse NAFLD. Recently, the role of ferroptosis in NAFLD has been attracting increasing attention [141,142]. Several studies have indicated that vitamin E may have a beneficial effect on the treatment of NAFLD [143]. Specifically, it can reduce serum ALT levels and improve liver steatosis and inflammation in patients with NAFLD [144,145]. Consequently, vitamin E could be considered a potential candidate for the treatment of NAFLD. Moreover, vitamin E has been shown to prevent acute liver failure due to ferroptosis caused by acetaminophen [146]. It also alleviates intestinal tissue damage and inflammation in necrotizing enteritis by mitigating ferroptosis in regulatory T cells (Tregs) [147]. Neurodegenerative diseases are characterized by the progressive loss of neurons and their myelin sheaths, leading to a deterioration of neurological function over time, including Alzheimer’s disease (AD), Parkinson’s disease (PD), Huntington’s disease (HD), and amyotrophic lateral sclerosis (ALS), among others [148]. Inactivation of system Xc− has been observed in a variety of neurodegenerative diseases. For instance, Kashani et al. reported a reduced expression of the glutamate (Glu) transporter in the cerebral cortex of individuals with AD, which inhibits the Xc− system [148]. Down-regulation of the *Slc7a11* gene and reduced levels of GSH have been observed in the brains of patients with PD [149,150]. In addition, dysregulation of GSH and GSH-dependent enzymes has been identified in individuals with HD [150]. Inactivation of system Xc− or GSH deficiency is an important factor in causing ferroptosis. Vitamin E (αTOH) supplementation has been shown to protect neurons from oxidative stress-induced damage, thereby positively influencing the prevention and progression of neurodegenerative diseases [3]. Mechanistically, vitamin E protects against PUFA-induced cell death, particularly that induced by docosahexaenoic acid, by preventing lipid peroxidation (LPO) through inhibition of oxidation. The high affinity of α-TOH for unpaired electrons enables it to disrupt the chain reaction cascade associated with membrane LPO. During this process, α-TOH scavenges active free radicals primarily through hydrogen transfer reactions, resulting in the formation of non-free radical α-TOH [151]. Furthermore, vitamin E has been identified to play a significant role in inhibiting ALOX, a factor involved in iron-mediated sagging, thereby preventing various ferroptosis-associated diseases [152,153,154].

Notably, it has been reported that tocotrienols exhibit greater antioxidant potential than tocopherols, which may be attributed to their greater distribution in cell membranes and their more efficient interaction with lipid peroxyl radicals [155]. However, their oral bioavailability is lower than that of tocopherols. Therefore, current research on vitamin E’s role in anti-ferroptosis primarily centers on tocopherols, particularly in vitro studies.

### 3.6. Vitamin K

**Figure 6:**
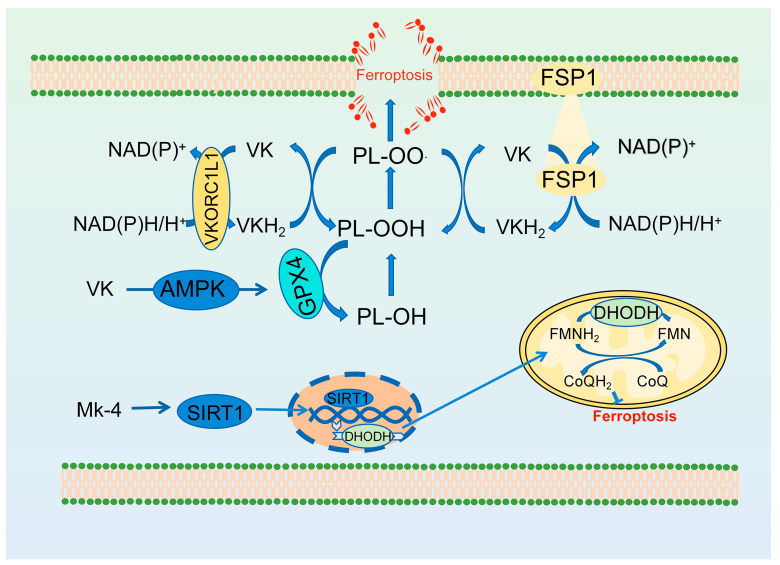
The anti-ferroptosis mechanisms of vitamin K. SIRT1: sirtuin 1; AMPK: AMP-activated protein kinase.

Vitamin K, a fat-soluble vitamin, is a general term for compounds with different structural forms, including VK_1_, VK_2_, VK_3_, and VK_4_ (Figure 6), which share the same menadione structure (i.e., the 2-methyl-1,4-naphthoquinone core) [156]. Our previous review of vitamin K presented a systematic analysis of the absorption, distribution, and metabolism of vitamin K [157]. Vitamin K is reduced by the action of VKORC1L1 to produce hydroquinone vitamin K, which serves as a cofactor to facilitate the activation of coagulation factors by gamma-glutamyl carboxylase (GGCX), thereby promoting blood clotting. Hydroquinone vitamin K (VKH_2_) is oxidized to VKO in this enzymatic reaction and is then reduced by vitamin K epoxide reductase complex subunit 1 (VKORC1) to produce vitamin K. Therefore, vitamin K is often used to stop bleeding or prevent bleeding in newborns [158]. In addition, Vitamin K plays an important role in maintaining bone and cardiovascular health because it can maintain the balance of mineralization in the body [157,159].

Kolbrink et al. first proposed that vitamin K is a potent inhibitor of ferroptosis and demonstrated its ability to mitigate ischemia-reperfusion acute kidney injury [160]. Following this, two significant studies further elucidated the underlying mechanisms of vitamin K anti-ferroptosis. Ito et al. found that vitamin K_2_ prevents ferroptosis in mouse fibroblasts and GPX4-deficient human cancer cell lines A375 and 786-O, as well as ferroptosis induced by the GPX4 inhibitor RSL3 [56]. Mechanistically, the researchers identified the reduced form, hydroquinone vitamin K, catalyzed by FSP1, as a direct trapping antioxidant of lipid free radicals, which reduces lipid peroxidation radicals to lipid peroxides (Figure 6) [56]. Furthermore, vitamin K demonstrated the capacity to mitigate organ ischemia-reperfusion injury by inhibiting ferroptosis and liver damage in Gpx4 knockout mice [56]. Yang et al. subsequently demonstrated that the classical vitamin K cycle pathway is involved in the anti-ferroptosis process [54]. They identified VKORC1L1, rather than VKORC1, as the key enzyme responsible for producing VKH_2_, which inhibits ferroptosis (Figure 6). Furthermore, their research determined the mechanism by which high concentrations of warfarin inhibit tumor growth and metastasis [54]. Warfarin induces ferroptosis in tumor cells that exhibit high levels of VKORC1L1 by inhibiting VKORC1L1 activity. In addition, several studies have reported that vitamin K can inhibit ferroptosis through non-vitamin K cycling pathways. Osteoarthritis is ameliorated by VK_2_ by suppressing ferroptosis through increasing GPX4 expression (Figure 6) [161]. Menaquinone-4 (MK4) inhibits ferroptosis in neuronal cells by activating sirtuin 1 (SIRT 1) to upregulate DHODH (Figure 6) [162]. This regulatory mechanism was also demonstrated again by Jin et al. [163], who reported that vitamin K_2_ inhibits ferroptosis in osteoblasts of diabetic mice through the activation of SIRT1, thereby alleviating osteoporosis. Furthermore, they found that AMPK is involved in the activation of SIRT 1 by vitamin K [163].

## 4. Discussion

There is a significant association between ferroptosis and the development and progression of various diseases, including diabetes, degenerative disorders, and tissue ischemic conditions [10]. Inhibiting ferroptosis through genetic strategies and pharmacochemical means could significantly inhibit the occurrence of these diseases [9,10]. Consequently, specific targeting of the key characteristics of ferroptosis, i.e., iron overload and lipid peroxidation, might represent a promising therapeutic strategy [9]. However, several ferroptosis inhibitors commonly used in animal or cell experiments remain far from clinical application. For example, desferriamine (DFO) has been approved by the U.S. Food and Drug Administration (FDA) for subcutaneous injection to alleviate elastin-induced ferroptosis in vitro [9]. However, it has a short half-life and induces agranulocytosis and other side effects [11,164]. Ferrostatin-1 (Fer-1) is the first synthetic ferroptosis inhibitor, which can stabilize free radicals and reduce ROS levels; however, it suffers from low stability and solubility [11]. Therefore, identifying compounds with low toxicity, wide sources, and high availability to resist ferroptosis is a research hotspot in the treatment of ferroptosis-related diseases.

Vitamins are essential trace elements that are widely distributed in nature and have been utilized to treat various diseases. For instance, vitamin A is employed to treat promyelocytic leukemia, vitamin D is used to prevent osteoporosis, and vitamin K is administered to prevent bleeding in newborns [165,166]. The administration of nutritional vitamin might be an effective way to regulate diseases associated with ferroptosis, which has garnered extensive research attention [17]. Importantly, in comparison with other exogenous ferroptosis inhibitors, vitamins offer advantages such as low cost and low toxicity. Few cases of systemic toxicity associated with natural vitamins have been reported in either animals or humans, except in circumstances of long-term high-dose intake. Additionally, the anti-ferroptotic properties of natural vitamins also provide a basis for the development of new anti-ferroptotic synthetic compounds. Improved analogs of vitamin E have been developed. For example, tetrahydronalidiol (THN) belongs to a specific group of azoles that are regarded as some of the most promising synthetic compounds to inhibit ferroptosis. Indeed, THN reacts with peroxy radicals almost 30 times faster than alpha-tocopherol. Hydronalidiol (HN), with an alkyl chain range of 12 to 15 carbons, protects cells from iron death more effectively than the classical ferroptosis inhibitors Fer-1 and liproxstatin-1 (Lip-1).

Greater attention must be directed toward several limitations in this field, although vitamins have made significant strides in anti- ferroptosis. (1) Vitamins have been extensively utilized in clinical to treat some diseases, including osteoporosis, night blindness, and bleeding. However, studies on vitamin-associated resistance to ferroptosis have primarily focused on cell experiments and animal models. There is a lack of large-scale, long-term clinical research with follow-up verification. (2) Compared with the iron chelation and lipid free radical capture type of ferroptosis inhibitors, the anti-ferroptosis mechanisms of some vitamins are complex. Vitamin A and vitamin D primarily exert an anti-ferroptosis effect by regulating the expression of antioxidant genes through their binding to specific nuclear receptors. Vitamin D receptors are mainly expressed in the thyroid, intestine, kidney, esophagus, lung, bone, and other tissues (Human Protein Atlas proteinatlas.org) [167]. As anticipated, vitamin D treatment was found to alleviate ferroptosis in the kidneys, intestines, bones, and other tissues. However, it is unknown whether vitamin D has an anti-ferroptosis effect in tissues with low VDR expression, which requires further verification. Additionally, while vitamin K can directly neutralize lipid radicals to prevent ferroptosis, the production of hydroquinone vitamin K necessitates the activities of specific reductases, such as FSP1 or VKORC1L1. However, VKORC1L1 is not widely expressed in a variety of cells. Therefore, the anti-ferroptosis effect of vitamins might only manifest in particular cell types, which limits their applicability in combating ferroptosis. (3) The issue of poor bioavailability presents a challenge to the clinical application of some vitamins. For example, vitamin E is nature’s most potent lipophilic radical-trapping antioxidant that protects against detrimental lipid peroxidation and ferroptosis, but its clinical application remains constrained due to its limited bioavailability and the high concentrations required for administration.

## 5. Conclusions

In general, vitamins have shown strong anti-ferroptosis abilities and the potential to treat ferroptosis-related diseases. Although several important mechanisms have been reported, the specific mechanism by which vitamins counteract ferroptosis remains unclear. This lack of clarity may limit the potential use of vitamins in the treatment of ferroptosis-related diseases. With the deepening of our understanding of ferroptosis, the ferroptosis-related mechanisms of vitamins will be further analyzed, leading to the expansion of the treatment of ferroptosis-related diseases using vitamins.
